# The Role of Poly-Herbal Extract in Sodium Chloride-Induced Oxidative Stress and Hyperlipidemia in Male Wistar Rats

**DOI:** 10.3390/medicines8060025

**Published:** 2021-05-31

**Authors:** Olubukola Sinbad Olorunnisola, Peter Ifeoluwa Adegbola, Bamidele Stephen Ajilore, Olayemi Adebola Akintola, Olumide Samuel Fadahunsi

**Affiliations:** 1 Department of Biochemistry, Faculty of Basic Medical Sciences, Ladoke Akintola University of Technology, Ogbomoso P.M.B 4000, Oyo State, Nigeria; osolorunnisola@lautech.edu.ng (O.S.O.); piadegbola27@lautech.edu.ng (P.I.A.); 2 Department of Medical Biochemistry, Osun State University, Osogbo P.M.B 4494, Osun State, Nigeria; bamidele.ajilore@uniosun.edu.ng; 3 Department of Science Laboratory and Technology, Faculty of Pure of Applied Sciences, Ladoke Akintola University of Technology, Ogbomoso P.M.B 4000, Oyo State, Nigeria; aoakintola@lautech.edu.ng

**Keywords:** sodium chloride, poly-herbal, antioxidants, lipid profile, liver, kidney, aorta

## Abstract

Consistent consumption of high salt diet (HSD) has been associated with increased cellular generation of free radicals, which has been implicated in the derangement of some vital organs and etiology of cardiovascular disorders. This study was designed to investigate the combined effect of some commonly employed medicinal plants on serum lipid profile and antioxidant status of aorta, kidney, and liver of high salt diet-fed animals. Out of the total fifty male Wistar rats obtained, fifteen were used for acute toxicity study, while the remaining thirty-five were divided into 5 groups of 7 animals each. Group 1 and 2 animals were fed normal rat chow (NRC) and 16% high salt diet (HSD) only, respectively. Animals in groups 3, 4 and 5 were fed 16% HSD with 800, 400, and 200 mg/kg bw poly-herbal extract (PHE), respectively, once for 28 consecutive days. Serum low-density lipoprotein (LDL), triacylglycerol (TG), total cholesterol (TC) and high-density lipoprotein (HDL), malondialdehyde, nitric oxide, catalase, superoxide dismutase, glutathione peroxidase, glutathione concentration, and activities were assessed in the aorta, kidney, and liver. Poly-herbal extract (*p* < 0.05) significantly reduced malondialdehyde and nitric oxide concentrations and also increased antioxidant enzymes and glutathione activity. Elevated serum TG, TC, LDL, and TC content in HSD-fed animals were significantly (*p* < 0.05) reduced to normal in PHE-treated rats while HDL was significantly elevated (*p* < 0.05) in a concentration-dependent manner in PHE treated animals. Feeding with PHE attenuated high-salt diet imposed derangement in serum lipid profile and antioxidant status in the organs of the experimental rats.

## 1. Introduction 

Nutrition is an important factor in maintaining the physiological and biochemical wellness of the biological system [1,2]. Constant consumption of diet deficient or excessive in micronutrients is associated with the development of degenerative and metabolic disorders [3]. Sodium chloride (NaCl) is probably the oldest spice in human history and has a multifunctional role in the modern-day food industry and biotechnology [4]. Recently, there has been a considerable increase in the salt content of foods due to changes in human dietary habits vis-à-vis high consumption of industrialized, processed, and fast foods [5,6,7], although, governmental and institutional awareness on the reduction of sodium consumption and negative health implications of high salt intake are well disseminated and publicized [7,8,9,10,11]. However, industrial suitability, gustatory delights, salt addiction, and consumer’s acceptability are a few of the factors still influencing the continued demand, interest, and consumption of high salt diet (HSD) worldwide [12,13]. Sodium is involved in several trans-membrane and physiological processes and is dominantly supplied via dietary salt [14,15]. Unfortunately, uncontrolled and excessive consumption of salt has been linked to the development of cardiovascular disorders, endothelial dysfunction, and derangement in lipid metabolism [16,17,18]. Increased activities of reactive oxidative species (ROS), infiltration of immune cells, and glomerular hyper-filtration have been postulated as the likely mechanisms of high salt-induced renal damage and hypertension [18,19,20,21]. Natural products and plants with medicinal importance are highly coveted and sought after throughout the world [22,23,24,25]. Pharmacological activities vis-à-vis cardio-protective, anti-inflammatory, antioxidant, anti-cancer prowess of these botanicals have been documented and attributed to their different phytochemicals [26]. Traditionally, many of these plants are employed as a concoction of poly-herbal mixture in the management of various diseases. It is a common practice and accepted belief in folkloric medicine that combination of herbal plants would have a rapid and potentiated effect against the targeted ailment [27,28].

*Annona muricata, Carica papaya, Moringa oleifera,* and *Aloe barbadensis* are medicinal plants with high ethno-botanical citations and are mostly and frequently employed as poly-herbal recipe in folkloric management of cardiovascular disorders in Nigeria and Africa [29]. Furthermore, numerous antioxidant and anti-hyperlipidemic activities of these four plants are well documented and cited in literature [30,31,32]. With this background understanding, this study was designed at investigating the efficacy of the combined aqueous leaf extract of these aforementioned plants against high salt diet-induced oxidative stress and derangement in lipid profile in male Wistar rats.

## 2. Materials and Methods

### 2.1. Plant Source and Extraction

*Annona muricata, Carica papaya, Moringa oleifera,* and *Aloe barbadensis* were collected in Ogbomoso town (8°08′ N 4°15′ E) and were authenticated by a taxonomist from the Department of Pure and Applied Biology, Ladoke Akintola University of Technology, Ogbomoso, Oyo State, Nigeria. The voucher numbers of the plants were deposited at the University Herbarium.

### 2.2. Preparation of Poly-Herbal Extracts (PHE)

Five hundred (500 g) each of the fresh leaves of the four plants were pulverized using kitchen blender (EMEL: EM-242, Shanghai, China) and subsequently macerated and boiled in water at 100 °C degrees for 2 h. It was left alone to cool under room temperature for 3 h and was then filtered using a clean muslin cloth. The supernatant was freeze-dried, and the lyophilized crude extract was stored in an airtight dark bottle and refrigerated until further use.

### 2.3. Acute Toxicity and Determination of LD_50_

Acute toxicity testing was carried out with fifteen animals in two phases according to the method described by Lorke [33].

### 2.4. Animal Grouping and Experimental Design

All animal procedures in this study were performed according to the guidelines of the research and ethics committee, Ladoke Akintola University of Technology (LAUTECH) for the use of laboratory animals. Ethical approval number LFBMSEC/26/2020 was collected from Faculty of Basic Medical Science Ethical Committee, LAUTECH, on the 4th of February, 2020.Thirty-five (35) healthy male Wistar rats weighing 140–150 g were obtained from the animal house of the Department of Biochemistry, College of Basic of Medical Sciences, LAUTECH. They were housed in ventilated cages on a 12:12 h light-dark cycle and acclimatized for 2 weeks and were randomly separated into 5 groups of 7 animals each as depicted in Table 1. Different concentrations of the extract were administered to the rats by oral gavage using a cannula.

### 2.5. Collection of Blood Serum and Tissue Preparation from Treated Rats 

The animals were sacrificed through cervical dislocation on the 29th day after overnight fasting. Blood was collected through cardiac puncture using a 5 mL syringe and transferred into plain sample bottles. The blood samples were centrifuged at 4000 rpm for 10 min to obtain the serum. The kidney, liver, and aorta were excised, washed in cold washing buffer, and homogenized in phosphate buffer (10% *w*/*v*). The homogenates were centrifuged at 10,000× *g* force for 15 min at 4 °C. The supernatants were collected and stored in the freezer at −18 °C.

### 2.6. Antioxidant Assays

Antioxidant enzyme activity and oxidative stress markers were estimated in the liver, aorta, and kidneys homogenates.

### 2.7. Determination of Superoxide Dismutase Activity

Superoxide dismutase (SOD) activity was evaluated according to the method of Misra and Fridovich [34], and 1.5 mL each of 75 mM of Tris-HCl buffer (pH 8.2), 30 mM EDTA, and 2 mM of pyrogallol were added to 70 µL of tissue homogenate. Change in absorbance was recorded at 420 nm for 3 min in a spectrophotometer.

### 2.8. Determination of Glutathione Concentration 

Glutathione (GSH) activity was estimated according to the procedure of Ellman [35]. One hundred (100 μL) of the tissue homogenate was diluted in 20 mL of phosphate buffer (0.1 M, pH 8). Fourty (40 mL) of (0.01 M) 5,5’-dithiobis (2-nitrobenzoic acid) (DTNB) (Sigma-Aldrich, Inc., Darmstadt, Germany) was added to 6 mL of the mixture, and absorbance was read at 412 nm.

### 2.9. Determination of Glutathione Peroxidase Activity

Glutathione peroxidase activity was determined according to the method of Reddy et al. [36]. To 3.0 mL of glutathione peroxidase substrate solution, 0.1 mL of the homogenate was added. To the test cuvette, 0.5ml of hydrogen peroxide was added and mixed. The change in absorbance was recorded every 30 s for 3 min in a spectrophotometer at 430 nm.

### 2.10. Determination of Catalase Activity

Catalase activity was determined according to the method of Clairborrne [37]. The reaction mixture contained 50 mM potassium phosphate buffer (pH 7.4), 19 mM H_2_O_2_, and 20 uL tissue homogenate. The degradation of H_2_O_2_ was read spectrophotometrically at 240 nm for 1 min and the catalase activity was calculated according to the formula K = 2.303/T × log (A1/A2), where K: Rate of reaction; T: Time interval (minutes); A1: Absorbance at time zero; A2: Absorbance at 60 s interval.

### 2.11. Determination of Malondialdehyde Concentration 

Estimation of malondialdehyde (MDA) concentration as an index of lipid peroxidation was assayed according to the method described by Ohkawa et al. [38], and 1 mL of 20% trichloroacetic acid (Sigma-Aldrich, Inc., Darmstadt, Germany) was added to 1 mL of the tissue homogenate thereafter 2 mL of 0.67% thiobarbituric acid (Sigma-Aldrich, Inc., Darmstadt, Germany) was added. The mixture was incubated at 100 °C for 15 min in a water bath and cooled. Six (6) mL of n-butanol was added and centrifuged at 3000 rpm for 15 min. The absorbance of the clear pink supernatant was then read against a blank at 532 nm spectrophotometrically. The concentration of MDA is expressed in nmol/g of the tissue.

### 2.12. Determination of Nitric Oxide Concentration

The level of nitric oxide (NO) in the tissues was determined according to the method described by Tsikas [39]. Succinctly, 200 μL of the homogenates was incubated with 200 μL of Griess reagent (Sigma-Aldrich, Inc., Darmstadt Germany) at 25 °C in the dark for 30 min. Absorbance was subsequently read at 548 nm.

### 2.13. Serum Lipid Assay 

Collected serum samples were analyzed for lipid profile. High-density lipoprotein-cholesterol (HDL-C) was assayed using an assay kit (Elabscience, Houston, TX, USA). Triglyceride (TG) content was evaluated by enzymatic method using an assay kit (RANDOX, Diagnostic, Crumlin, Ireland). Total cholesterol (TC) was determined according to the method of Parakh and Jank [40]. Low-density lipoprotein (LDL-C) and Very low-density lipoprotein—cholesterol (VLDL-C) was calculated according to Friedwald et al. [41].

### 2.14. Statistical Analysis

Data obtained in this study were expressed as mean ± SEM and subjected to one-way analysis of variance (ANOVA) using statistical package for social sciences 21.0. Duncan’s multiple test was used to identify significance between means at *p* < 0.05.

## 3. Results 

### 3.1. Acute Toxicity Studies of PHE Extracts

Firstly, nine animals were randomly divided into three groups of three animals each. Each group of animals was administered 10, 100, and 1000 mg/kg of the poly-herbal extract, and no mortality was observed. In the second phase, 6 animals were distributed into 3 groups of two animals each and were administered higher 1600, 2900, and 5000 mg/kg of the PHE, respectively. No mortality was observed after 24 to 48 h among animals in all groups.

### 3.2. Antioxidant Status of the Liver 

**Figure 1:**
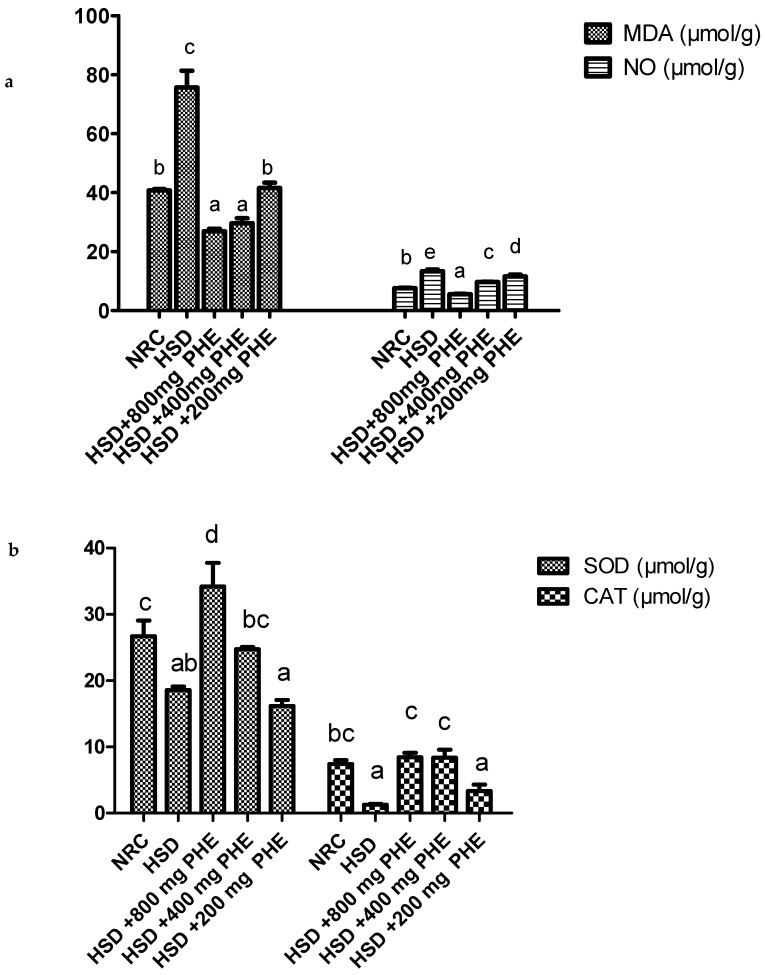
(**a**) Malondialdehyde (MDA) and Nitric oxide (NO) concentration in the liver of rats. (**b**) Super oxide dismutase (SOD) and catalase (CAT) activity in the liver of rats. Data were expressed as mean ± SEM. Bar charts with different alphabets are significantly (*p* < 0.05) different. NRC: normal rat chow; HSD: high salt diet; PHE: poly-herbal extract. Figure 1b revealed that SOD and CAT activities were reduced in the liver of the high salt fed (HSD) rats and were significantly (*p* < 0.05) increased after treatment with 800 and 400 mg/kg poly-herbal extract (PHE).

The antioxidant enzyme activities and concentration of oxidative stress markers in the liver of rats in all experimental groups are depicted in Figure 1a,b and Table 2.

Figure 1a shows that malondialdehyde (MDA) and nitric oxide concentration in the liver was significantly (*p* < 0.05) elevated in HSD only fed group. Poly-herbal extract at 800, 400 and 200 mg/kg caused a significant (*p* < 0.05) and dose-dependent reduction in MDA concentration, while NO concentration was only significantly (*p* < 0.05) decreased at 800 and 400 mg/kg PHE treatment.

In Table 2, it was observed that the concentrations of GSH, GP_x_, and GST were reduced in HSD only treated animals. The PHE at 800 mg/kg significantly elevated GSH concentration, while GPx and GST activities were significantly (*p* < 0.05) elevated by treatment with 800 and 400 mg/kg PHE, respectively.

### 3.3. Antioxidant Status of the Kidney 

**Figure 2:**
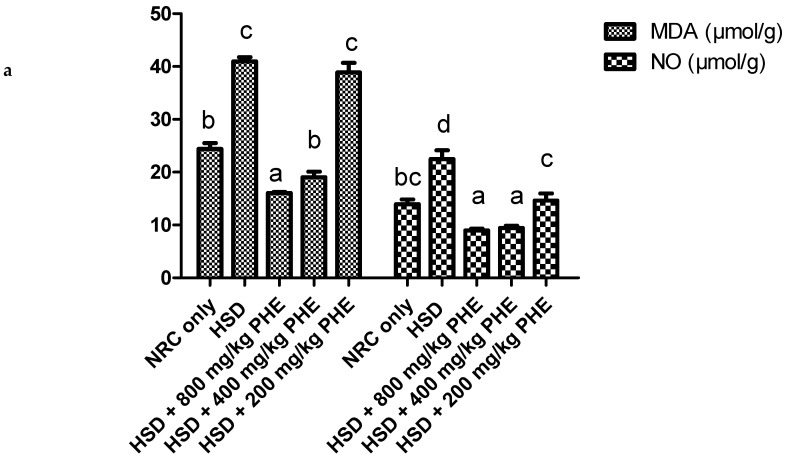

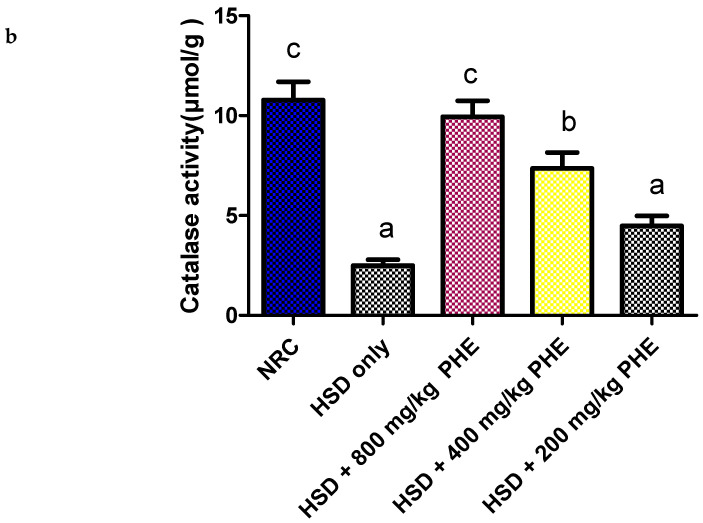
(**a**) Malondialdehyde (MDA) and Nitric oxide (NO) concentration in the liver of rats. (**b**) Catalase activity in the kidney of rats. Bar charts with different alphabets are significantly (*p* < 0.05) different. NRC, normal rat chow; HSD, high salt diet; PHE: poly-herbal extract. The concentration of GSH and activities of GPx and GST were significantly (*p* < 0.05) reduced in the kidneys of HSD only fed rats Table 3. However, treatment with 800 and 400 mg/kg PHE significantly (*p* < 0.05) elevated the activities of these proteins.

The antioxidant enzyme activities and concentration of oxidative stress markers in the kidney of rats in all experimental groups are depicted in Figure 2 and Table 3. It was observed in Figure 2a that malondialdehyde and nitric oxide concentration were significantly (*p* < 0.05) elevated in HSD only fed group. However, a significant (*p* < 0.05) and dose-dependent reduction in the concentration of MDA and NO was observed after treatment with 800 and 400 mg/kg PHE.

The activity of catalase in the kidney of experimental rats is depicted in Figure 2b. High salt diet (HSD) caused a significant (*p* < 0.05) reduction in the activity of catalase in the kidney of treated rats. However, a dose dependent and significant (*p* < 0.05) increase in catalase activity was observed after treatment with 800, 400, and 200 mg/kg PHE extract, respectively.

### 3.4. Antioxidant Status of the Aorta 

**Figure 3:**
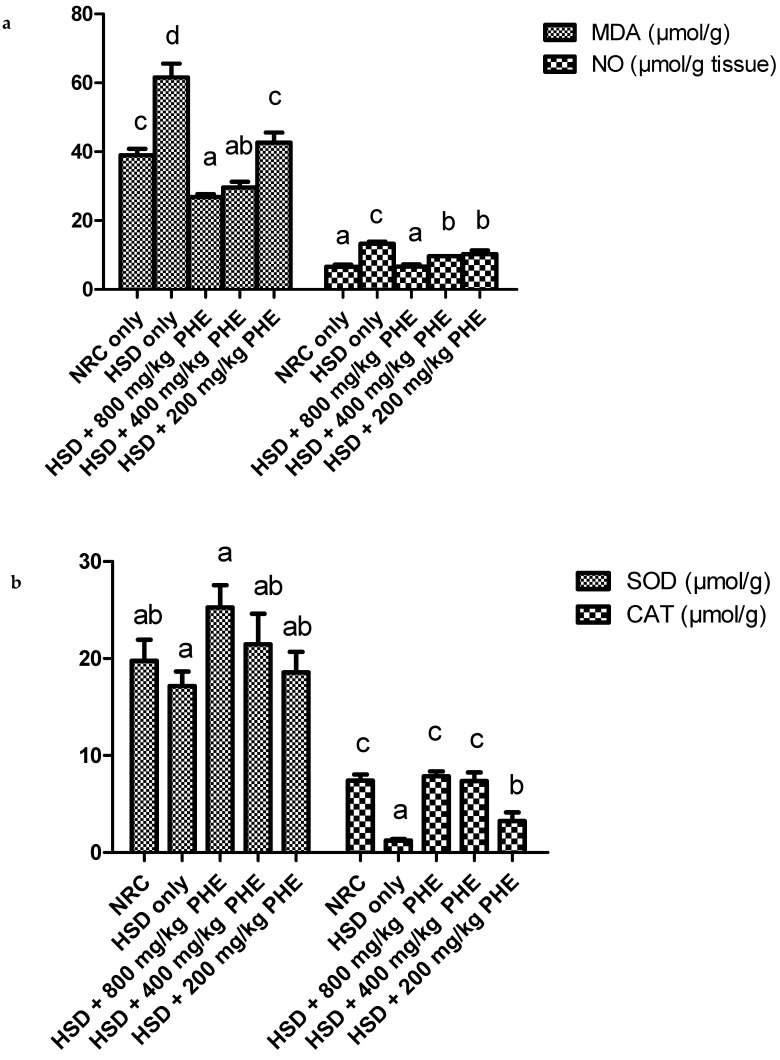
(**a**) MDA and NO activities in the aorta of rats. (**b**) SOD and CAT activities in the aorta of rats. Bar charts with different alphabets are significantly (*p* < 0.05) different. NRC, normal rat chow; HSD, high salt diet; PHE: poly-herbal extract.

Antioxidant enzyme activities and concentration of oxidative stress markers in the aorta of rats in all experimental groups are depicted in Figure 3 and Table 4.

Figure 3a depicts that malondialdehyde and nitric oxide concentration in the aorta of HSD only fed rats was significantly (*p* < 0.05) higher relatively to other groups. Administration with 800, 400, and 200 mg/kg PHE significantly decreased MDA and NO levels in the aorta of treated rats.

The aortic activities of catalase and superoxide dismutase enzyme are shown in Figure 3b. A significantly (*p* < 0.05) lowered activity was observed in the HSD only fed rats when compared with other groups. Administration of PHE at 800 and 400 mg/kg significantly (*p* < 0.05) elevated the activity of aortic catalase and superoxide in treated rats.

Table 4 indicates the activities of GSH, GPx, and GST in the aorta of rats. It was noticed that GSH, GPx, and GST concentration was considerably reduced in HSD on fed animals. However, the activities of these proteins in aorta of rats were notably (*p* < 0.05) elevated in the 800 and 400 mg/kg PHE treated rats.

### 3.5. Serum Lipid Profile of Treated Rats

HDL and LDL levels in the serum of rats are depicted in Table 5. Respectively, HDL concentration was significantly (*p* < 0.05) depleted, while LDL levels were significantly (*p* < 0.05) elevated in the HSD exposed rats. The 800 and 400 mg/kg PHE treated rats showed significant (*p* < 0.05) elevated HDL, with a concomitant and significantly (*p* < 0.05) reduced LDL concentration.

**Figure 4:**
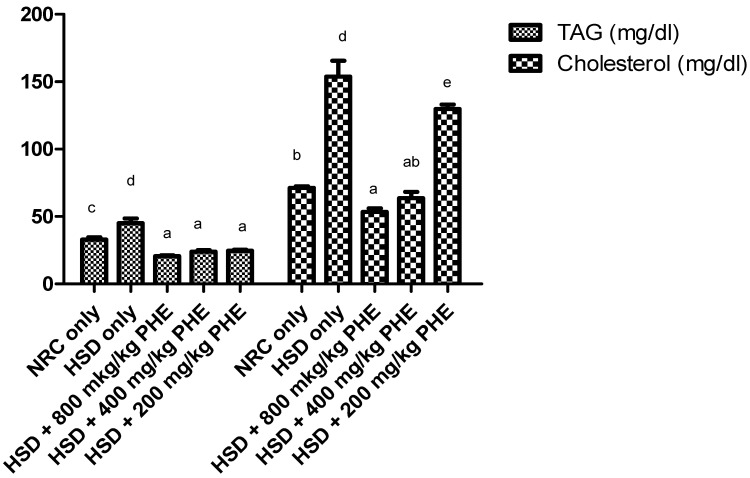
Serum triacylglycerol and cholesterol concentration in the serum of rats. Data were expressed as mean ± SEM. Bar charts with different alphabets are significantly different (*p* < 0.05).

Triacylglycerol and cholesterol concentration in the serum of treated rats is shown in Figure 4. It was noted that triacylglycerol and cholesterol concentration in the serum of the HSD only fed rats were significantly (*p* < 0.05) elevated when compared with other groups of experimental animals. There was a dose-dependent and significant (*p* < 0.05) decrease in serum TAG concentration after treatment with PHE. An appreciable and more pronounced reduction in cholesterol concentration was observed in the serum of 800 and 400 mg/kg PHE treated animals.

### 3.6. Gas Chromatography Mass Spectrophotometry (GC-MS) Analysis 

**Figure 5:**
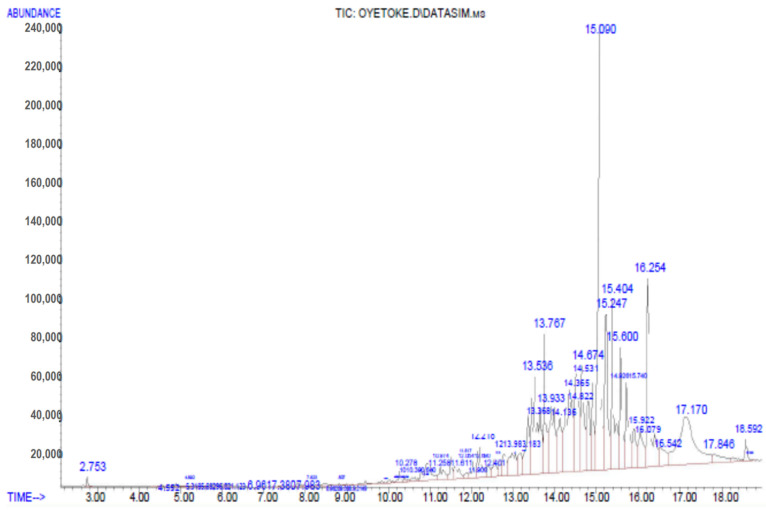
GC–MS chromatogram of the investigated poly-herbal extract.

GC-MS spectrum of PHE with peaks and retention time is shown in Figure 5. The analysis of the poly-herbal extract revealed the presence of about 61 compounds (Table 6) with compounds such as Benzene-2-tert-butyldimethylsilyloxy]-1-isopropyl-4-methyl- (8.83%), Deoxyqinghaosu (8.46%), Benzene, 1,1’-(1,2-cyclobutanediyl)-bis-,trans- (5.15%), N-2-Acetylcyclopentylidene-cyclohexylamine (4.18%), 9,10-Anthraquinone-monohydrazone (3.83%), scopoletin (3.66%), 2,3-Diphenylcyclopropyl-methylphenylsulfoxide (3.59%), and Bicyclo [3.3.1]nonan-2-one,1-methyl-9-(1-methylethylidene (2.45%) detected to be notably present.

## 4. Discussion

Excessive consumption of dietary salt has been associated with increase production of free radicals which can overwhelm cellular antioxidant and defense mechanism [42,43]. The deleterious effect and consequences of free radicals on vital organs have been clinically and experimentally established [44]. Reactive oxygen species when not appropriately regulated and/or quenched oxidize important biological molecules in tissues [43,45]. The kidney, liver, and heart are important organs that are central to the metabolic processes of the biological system. Hence, an oxidative insult to these organs will have a negative effect on overall cellular homeostasis. Considerable elevated MDA and NO levels noted in the liver, kidney, and aorta homogenates of animals fed with 16% high salt diet without treatment indicate that there was a significant increase in lipid peroxidation and oxidation in these tissues relative to the NRC and 400 and 800 mg/kg PHE groups, hence suggesting a protective effect of the poly-herbal extract used in this study. Malondialdehyde is a product of membrane lipid peroxidation resulting from the harmful effect of superoxide anion (*O_2_), hydroperoxyl radicals (HO∙_2_), lipid radicals (*L), peroxy-radical (*LOO), and peroxynitrite (ONOO-) [46,47]. The consequence of this is a considerable distortion in the conformation, physiological architecture, and integrity of the membrane as any major alteration and oxidation of the membrane lipids might have significant and negative aftermath on the signaling capacity and process of the cell [46,48,49]. Nitric oxide is a free radical which is generated as an immunological response in many cell types [50,51]. Although, there are contrasting reports on its clinical significance, however, increased concentration of different nitric oxide isoforms has been reported to manifest in cardiac and vascular diseases [52,53].

High salt diet significantly reduced the SOD, CAT, and selenocysteine peroxidase in the liver, kidney, and aorta HSD only fed red rats. No major antioxidant improvement in these organs was noticed at the lowest dose of the extract but was more buoyed and pronounced at higher dosages (400 and 800 mg/kg) of the PHE treatment.

Activities of enzymic antioxidants are useful indices and markers in the prognosis, progression, and prediction of some disease conditions [54]. Superoxide dismutase, catalase, and glutathione peroxidase are first-line defense antioxidants shielding the body against dangerous radicals vis-à-vis superoxide anion, peroxisomal, and mitochondrial hydrogen peroxide respectively [54].

Glutathione S-transferases catalyze the nucleophilic attack of glutathione (GSH) on electrophilic substrates, thereby decreasing their reactivity with cellular macromolecules [54]. Glutathione has many functions in the mammalian cell among which is the elimination and protection against reactive nitrogen and oxygen species [55]. In this present study, high salt diet reduced the concentration of GSH in the salt-treated animals, although this is not statistically different from the normal chow fed rats. GSH was only significantly elevated in 800 mg/kg extract-treated animals. The antioxidants depletion effect of high salt diet recorded in this study is in unison with previous scientific submission of [56]. As established by Bayorh et al. [57] and Saidu et al. [58], the activity of antioxidant enzymes decreased, while ROS and MDA concentration increased in rats fed with 8% salt diet for 3 weeks. Batteries of experimental reports have documented that high sodium chloride can elicit derangement in lipid metabolism [59,60,61]. In this study, cholesterol, triglyceride and low-density lipoprotein which are predictors of cardiovascular disorders [62] were elevated, while HDL level was reduced in the serum of salt-loaded rats. Nonetheless, the concentration of these markers was reversed to near normal after treatment with PHE. Oxidative stress has been reported to play a role in the derangement of lipid homeostasis through oxidation of accumulated low-density lipoprotein cholesterol in the plasma. This has been implicated in the development of atherosclerosis and heart attack [63]. The crude extracts and fractions of plants employed in this study have been discerned to contain different phenolics and important secondary metabolites such as caffeic acid, rutin, kaempferol, chlorogenic acid, procyanidins, catechin, and epicatechin with documented pharmacological and biological activities [64,65]. Some empirical pieces of evidence have reported various extracts of the plants used in this study to demonstrate substantial anti-hyperlipidemic activities in experimentally induced pathological states [66,67,68]. For instance, *Aloe barbadensis, A.muricata,* and *M. oleifera* have been reported to modulate lipase activity and increase the activity hormone sensitive lipase (HSL) resulting in improved lipid profile [30]. Different mechanisms including amelioration of deranged amino acid and pyrimidine metabolism, inhibition of HMG-CoA reductase, and enhancement of lipid oxidation through activation of adenosine monophosphate-activated protein kinase (AMPK) pathway by active principles in these plants have been postulated [69,70]. Polyphenols such as flavonoids and phytosterols which are available in the individual extract of PHE used in this study have been reported to ameliorate derangement in lipid homeostasis by activating peroxisome proliferator activated receptors (PPAR) and modulate redox signaling pathways and anti-oxidant system by inhibiting xanthine oxidase and also inducing transcription factors such as nuclear erythroid factor (Nrf2) which subsequently binds to antioxidant regulatory elements (ARE), thus initiating the expression of cytoprotective and antioxidant genes, leading to enhanced synthesis of enzymatic antioxidants [31,71,72,73,74,75]. Furthermore, active principles elicit antioxidant potential by donating and transferring hydrogen atom and single electron to free radicals, thus disrupting their deleterious impact in the body [76]. It is worthy to note that compounds belonging to important classes of secondary metabolites vis-à-vis alkaloids, flavonoids, and terpenoids were recognized and detected in the chromatographic analysis. Active principles such as coumarin, scopoletin, and isoopulegol were detected in this study and have been documented to evoke antioxidant, inflammatory, anti-hyperlipidemic, and anti-bacteria activities [76,77,78,79,80,81,82]. Furthermore, isolated anthraquinones from different medicinal plants have exhibited in vitro radical scavenging potential [83,84], while deoxyqinghaosu and corydaldine identified in this study have also been acclaimed to display arrays of biological activities [84].

## 5. Conclusions

High salt diet exposure elicited derangement in the antioxidant status in the assessed organs of the experimental rats. However, treatment with the different concentrations of the poly-herbal extracts caused a considerable decrease in pro-oxidative stress markers and increased the antioxidant proteins with improved serum lipid profile.

## Figures and Tables

**Table 1:** Animal grouping and experimental design.

Groups	Treatment
1	Fed with normal rat chow only (positive control)
2	Fed with 16% salt diet only (negative control)
3	Fed with 16% salt diet and 800 mg/kg of the poly-herbal extract once daily
4	Fed with 16% salt diet and 400 mg/kg of the poly-herbal extract once daily
5	Fed with 16% salt diet and 200 mg/kg of the poly-herbal extract once daily

**Table 2:** Concentration of GSH, GPx, and GST in the liver of rats.

Treatment	GSH (µmol/g)	GP_X_ (µmol/g)	GST (µmol/g)
NRC	2.77 ± 0.30 ^ab^	0.63 ± 0.04 ^bc^	5.90 ± 0.91 ^b^
HSD only	2.29 ± 0.44 ^ab^	0.55 ± 0.01 ^ab^	3.34 ± 0.54 ^a^
HSD + 800 mg/kg PHE	3.25 ± 0.38 ^b^	0.81 ± 0.07 ^d^	6.37 ± 0.81 ^b^
HSD + 400 mg/kg PHE	2.67 ± 0.08 ^ab^	0.73 ± 0.01 ^cd^	5.66 ± 0.55 ^b^
HSD + 200 mg/kg PHE	2.04 ± 0.23 ^a^	0.42 ± 0.05 ^a^	4.34 ± 0.54 ^ab^

Data were expressed as mean ± SEM. Values with different superscripts down the column are significantly different (*p* < 0.05). NRC: normal rat chow; HSD: high salt diet; PHE: poly-herbal extract.

**Table 3:** Concentration of GSH, GPx, and GST in the kidney of rats.

Treatment	GSH (µmol/g)	GP_X_ (µmol/g)	GST (µmol/g)
NRC only	1.61 ± 0.25 ^bc^	0.55 ± 0.02 ^b^	2.41 ± 0.39 ^b^
HSD only	0.91 ± 0.04 ^a^	0.31 ± 0.04 ^a^	0.50 ± 0.13 ^a^
HSD + 800 mg/kg PHE	1.68 ± 0.15 ^bc^	1.09 ± 0.03 ^d^	2.41 ± 0.39 ^b^
HSD + 400 mg/kg PHE	1.50 ± 0.16 ^bc^	0.76 ± 0.05 ^c^	1.39 ± 0.13 ^ab^
HSD + 200 mg/kg PHE	1.09 ± 0.02 ^ab^	0.76 ± 0.06 ^c^	0.50 ± 0.01 ^a^

Data were expressed as mean ± SEM. Values with different superscripts down the column are significantly different (*p* < 0.05). NRC: normal rat chow; HSD: high salt diet; PHE: poly-herbal extract.

**Table 4:** GSH, GPx, and GST activities in the aorta of rats.

Treatment	GSH (µmol/g)	GP_X_ (µmol/g)	GST (µmol/g)
NRC only	2.77 ± 0.3 ^ab^	0.63 ± 0.04 ^b^	5.90 ± 0.91 ^b^
HSD only	1.93 ± 0.25 ^a^	0.51 ± 0.03 ^a^	3.34 ± 0.54 ^a^
HSD + 800 mg/kg PHE	3.25 ± 0.38 ^b^	0.71 ± 0.04 ^b^	6.37 ± 0.81 ^b^
HSD + 400 mg/kg PHE	2.67 ± 0.08 ^ab^	0.66 ± 0.05 ^b^	5.61 ± 0.55 ^b^
HSD + 200 mg/kg PHE	2.03 ± 0.23 ^a^	0.68 ± 0.04 ^b^	4.34 ± 0.54 ^ab^

Data were expressed as mean ± SEM. Values with different superscripts down the column are significantly different (*p* < 0.05). NRC: normal rat chow; HSD: high salt diet; PHE: poly-herbal extract.

**Table 5:** Concentration of high-density lipoprotein (HDL) and low-density lipoprotein (LDL) in the serum of rats.

Treatment	HDL (mg/dL)	LDL (mg/dL)
NRC only	6.63 ± 0.43 ^ab^	35.06 ± 1.97 ^c^
HSD only	4.91 ± 0.85 ^a^	163.29 ± 1.59 ^f^
HSD + 800 mg/kg PHE	22.83 ± 0.72 ^d^	9.67 ± 1.67 ^a^
HSD + 400 mg/kg PHE	21.99 ± 0.64 ^d^	27.25 ± 2.69 ^b^
HSD + 200 mg/kg PHE	12.09 ± 1.20 ^c^	89.30 ± 3.76 ^e^

NRC: normal rat chow; HSD: high salt diet; PHE: poly-herbal extract. Values with different superscripts down the column are significantly different (*p* < 0.05). NRC: normal rat chow; HSD: high salt diet; PHE: poly-herbal extract.

**Table 6:** Compounds detected in the poly-herbal extract using GC–MS analysis.

Retention Time		Identified Compounds	Peak Area %
1	2.651	Arsenous acid, tris(trimethylsilyl)ester	0.09
2	2.905	2,4-Cyclohexadien-1-one	0.07
3	3.271	Cyclotrisiloxane, hexamethyl-	0.21
4	3.834	1,4-Bis(trimethylsilyl)benzene	1.18
5	4.341	Tris(tert-butyldimethylsilyloxy)arsane	0.05
6	4.398	1,1,1,3,5,5,5-Heptamethyltrisiloxane	0.06
7	4.764	4-Methyl-2-trimethylsilyloxy-acetophenone	0.13
8	4.905	1,3,5,7-Cyclooctatetraene	0.39
9	5.327	Cyclotetrasiloxane, octamethyl-	0.14
10	5.440	Trans-4-Dimethylamino-4’-methoxych alcone	0.39
11	5.834	1,1,3,3,5,5,7,7-Octamethyl-7-(2-methylpropoxy) tetrasiloxan-1-ol	0.26
17	6.961	1,1,1,3,5,5,5Heptamethyltrisiloxane	0.05
18	7.158	Arsenous acid, tris(trimethylsilyl) ester	0.04
19	7.468	1,2-Bis(trimethylsilyl)benzene	0.11
20	7.722	1,1,1,3,5,5,5Heptamethyltrisiloxane 1H-Indole	0.04
22	8.510	Cyclopentasiloxane, decamethyl-	1.22
23	8.736	5-Methyl-2-phenylindolizine	0.12
24	9.017	3,3-Diisopropoxy-1,1,1,5,5,5-hexam ethyltrisiloxane.	0.05
25	9.186	1,2,4Triazolo[1,5-a]pyrimidine-6-carboxylic acid	0.04
26	9.327	Silane	0.10
27	9.863	Octasiloxane, 1,1,3,3,5,5,7,7,9,9, 11,11,13,13,15,15-hexadecamethyl-	0.23
28	10.144	4-Bromo-3-chloroaniline	0.32
29	10.285	Cyclohexasiloxane, dodecamethyl-	1.47
30	10.651	Heptasiloxane, 1,1,3,3,5,5,7,7,9,9,11,11,13,13-tetradecamethyl-	0.54
31	10.961	Alpha.-D-Ribofuranoside ((2-pyridy l)-2,3-O-isopropylidene-1-thio-	1.28
32	11.384	Coumarin	1.01
33	11.609	Cycloheptasiloxane, tetradecamethy1-	1.85
34	11.947	Anthracene, 9,10-diethyl-9,10-dihydro-	0.38
35	12.313	3-Quinolinecarboxylic acid, 6,8-di fluoro-4-hydroxy-, ethyl ester	1.15
36	12.595	2-Ethylacridine	0.74
37	12.680	Cyclooctasiloxane, hexadecamethyl-	1.02
38	12.792	5,5’-Di(ethoxycarbonyl)-3,3’-dimethyl-4,4’-dipropyl-2,2’-dipyrrylmethane	0.97
39	13.018	Trans-3-Ethoxy-b-methyl-b-nitrostyrene	1.58
40	13.187	Corydaldine	1.11
41	13.356	Benzene, 1,1’-(1,2-cyclobutanediyl)bis-,trans-	5.15
42	13.778	Isopulegol	1.78
43	13.947	Bicyclo[4.1.0]hepta-2,4-diene, 2,3,4,5-tetraethyl-7,7-diphenyl-	2.93
44	14.144	2-Methyl-7-phenylindole	2.10
45	14.398	1H-Indole-2-carboxylic acid, 6-(4- ethoxyphenyl)-3-methyl-4-oxo-4,5,6,7-tetrahydro-, isopropyl ester	4.10
46	14.539	Scopoletin	3.66
47	14.680	Bicyclo[3.3.1]nonan-2-one,1-methyl-9-(1-methylethylidene)-	2.45
48	14.933	2,4,6-Trimethylphenyl isothiocyanate	1.79
49	15.074	Deoxyqinghaosu	8.46
50	15.271	N-(2-Acetylcyclopentylidene)cyclohexylamine	4.18
51	15.412	Fluorenoneoxime	2.58
52	15.750	6-Methoxy-2-hydroxyquinoxaline-4-oxide	2.80
53	15.947	Benzo[h]quinoline, 2,4-dimethyl-	1.51
54	16.088	Tris(tert-butyldimethylsilyloxy)arsane Propiophenone, 2’-(trimethylsiloxy)-	1.87
55	16.257	9,10-Anthraquinone monohydrazone	3.83
56	16.426	1,2-Benzisothiazol-3-amine tms	2.02
57	17.215	Benzene, 2-[(tert-butyldimethylsil yl)oxy]-1-isopropyl-4-methyl-	8.83
58	17.863	1,2-Bis(trimethylsilyl)benzene	2.10
59	18.229	Tetrasiloxane, decamethyl- 1,4-Bis(trimethylsilyl)benzene	1.49
60	18.595	2,3-Diphenylcyclopropyl)methylphenylsulfoxide, trans-	3.59
61	18.764	Trimethyl[4-(2-methyl-4-oxo-2-pent yl)phenoxy]silane	0.62

## Data Availability

The datasets used and/or analyzed during the current study are available from the corresponding author on reasonable request.
